# Change in Tear Film Lipid Layer Thickness, Corneal Thickness, Volume and Topography after Superficial Cauterization for Conjunctivochalasis

**DOI:** 10.1038/srep12239

**Published:** 2015-07-17

**Authors:** Tommy C. Y. Chan, Cong Ye, Paul KF Ng, Emmy Y. M. Li, Hunter K. L. Yuen, Vishal Jhanji

**Affiliations:** 1 Department of Ophthalmology and Visual Sciences, The Chinese University of Hong Kong; 2 Hong Kong Eye Hospital, Mongkok, Kowloon, Hong Kong; 3 Centre for Eye Research Australia, University of Melbourne, Victoria, Australia

## Abstract

We evaluated the change in tear film lipid layer thickness, corneal thickness, volume and topography after superficial cauterization of symptomatic conjunctivochalasis. Bilateral superficial conjunctival cauterization was performed in 36 eyes of 18 patients with symptomatic conjunctivochalasis. The mean age of patients (12 males, 6 females) was 68.6 ± 10.9 years (range: 44–83 years). Preoperatively, 28 eyes (77.8%) had grade 1 conjunctivochalasis, and 8 eyes (22.2%) had grade 2 conjunctivochalasis. At 1 month postoperatively, the severity of conjunctivochalasis decreased significantly (p < 0.001) and 29 eyes (80.6%) had grade 0 conjunctivochalasis whereas 7 eyes (19.4%) had grade 1 conjunctivochalasis. The mean Ocular Surface Disease Index score decreased from 31.5 ± 15.2 preoperatively to 21.5 ± 14.2 at the end of 1 month postoperatively (p = 0.001). There was a statistically significant increase in mean tear film lipid layer thickness 1 month after the surgery (49.6 ± 16.1 nm vs 62.6 ± 21.6 nm; p < 0.001). The central corneal thickness, thinnest corneal thickness and corneal volume decreased significantly postoperatively (p < 0.001). Our study showed that superficial conjunctival cauterization is an effective technique for management of conjunctivochalasis in the short term. An increase in tear film lipid layer thickness along with a decrease in corneal thickness and volume were observed after surgical correction of conjunctivochalasis.

Conjunctivochalasis is a bilateral disorder characterized by folds of inferior bulbar conjunctiva overlying the lower eyelid margin. The redundant conjunctiva is associated with disruption of the tear meniscus^1^,^2^, delayed tear clearance and, ocular surface inflammation^3^,^4^,^5^. Symptoms of conjunctivochalasis include ocular irritation and dryness, epiphoria, and subconjunctival haemorrhage^6^,^7^. Symptomatic conjunctivochalasis can be treated with topical lubricants with or without topical corticosteroids. Non-responding cases can be managed with surgical excision of the conjunctiva with or without amniotic membrane transplantation^8^,^9^,^10^. Recent studies have also demonstrated successful reduction of conjunctivochalasis with superficial cauterization^11^,^12^, radiofrequency ablation and laser photocoagulation^13^,^14^.

Conjunctivochalasis is associated with an unstable tear film, increased tear osmolarity and ocular surface inflammation^5^,^15^,^16^. These changes may further lead to alterations in the internal milieu of tear film complex. Although conjunctivochalasis is associated with changes in tear film dynamics, the diagnosis of this entity is mainly clinical. There is no previous study that has evaluated the physical characteristics of the tear film lipid layer or corneal parameters in cases with conjunctivochalasis. Ocular surface interferometry is a noninvasive method used for measurement of the tear film lipid layer thickness. It has been used for the evaluation of dry eye symptoms and meibomian gland dysfunction^17^,^18^,^19^,^20^,^21^. Colored fringes are generated from interference between light reflected from the surface of the lipid layer and from lipid-aqueous layer interface. It has been suggested that these interference patterns could be used to observe the fluidity and thickness of the lipid layer^22^,^23^. With growing knowledge of the precorneal tear film, lipid layer has been found to play an important role in the maintenance of tear film stability^24^,^25^. The present study aimed to assess the changes in tear film lipid layer thickness after superficial cauterization of symptomatic conjunctivochalasis. In addition, we investigated the effect of surgical correction of conjunctivochalasis on corneal thickness, volume and topography.

## Results

In normal subjects, the RC, CV, and ICC for ICU were 10.8 (95% confidence interval: 8.1–13.5), 6.6% (4.9%–8.3%), and 0.926 (0.836–0.972), indicating a high test-retest variability for ICU measurement.

The mean age of patients (12 males, 6 females) with conjunctivochalasis was 68.6 ± 10.9 years (range: 44–83 years). The patients were diagnosed with mild to moderate conjunctivochalasis, which were classified as Grade 1 (a single, small fold) to 2 (≥2 folds, not higher than the tear meniscus) on slit-lamp biomicroscopic examination^6^. Superficial cauterization was performed in all cases with no intraoperative or postoperative complications.

Preoperatively, 28 eyes (77.8%) had grade 1 disease, and 8 eyes (22.2%) had grade 2 disease. At 1 month postoperatively, 29 eyes (80.6%) had grade 0 conjunctivochalasis (absence of persistent folds) and 7 eyes (19.4%) had grade 1 disease (p < 0.001). The mean OSDI score (±standard deviation) was 31.5 ± 15.0 (range: 6.3 to 68.8) preoperatively and 21.5 ± 14.0 (range: 4.2 to 60.4) at the end of 1 month postoperatively (p < 0.001). The OSDI scores decreased in 15 patients (83.3%), remained unchanged in 2 patients (11.1%) and increased in 1 patient (5.6%). The severity of conjunctivochalasis also decreased significantly after the treatment (p < 0.001).

There was no significant change between the Schirmer’s test results before and after the treatment (p = 0.399) (Table 1). There was a statistically significant increase in the ICU one month after surgery (p < 0.001) from a mean of 49.6 ± 16.1  nm (range: 29 to 92) preoperatively to 62.6 ± 21.6 nm (range: 26 to 100) postoperatively. Notably, the ICU of control eyes was 58.9 ± 13.9 nm (range: 44 to 93), which was significantly higher than the preoperative ICU (p = 0.048) of patients with conjunctivochalasis. However, the ICU value of normal subjects was comparable to the postoperative ICU values of patients with conjunctivochalasis patients (p = 0.548) indicating restoration of lipid layer thickness after surgery.

The maximum keratometry values before and after superficial cauterization of conjunctivochalasis were 45.7 ± 2.0 D and 45.8 ± 1.9 D, respectively (p = 0.568). The average keratometry value increased from 44.0 ± 1.3 D preoperatively to 44.1 ± 1.3 D at 1 month (p = 0.085). The central corneal thickness, thinnest corneal thickness and corneal volume decreased significantly compared to the preoperative values (p < 0.001). The preoperative central corneal thickness and corneal volume were 553.5 ± 22.8 μm and 59.7 ± 2.5 mm^3^, respectively. The corresponding postoperative values were 547.6 ± 22.6 μm and 58.4 ± 2.5 mm^3^ respectively (Table 1).

## Discussion

Conjunctivochalasis is an ocular surface disease that presents with ocular irritation, dry eye symptoms and epiphora^6^. Surgical reconstruction of the tear meniscus in conjunctivochalasis leads to an improvement of ocular signs and symptoms^1^,^9^, tear film break up time, Schirmer’s test, fluorescence clearance test, tear osmolarity^15^,^26^,^27^, and tear film inflammatory markers^5^. Superficial conjunctival cauterization has been shown to be an effective treatment for correction of conjunctivochalasis^11^,^12^,^28^. Haefliger and coworkers reported that superficial cauterization of the inferior bulbar conjunctiva results in a significant reduction of moderate conjunctivochalasis^12^. Nakasato et al showed that thermocautery could induce conjunctival shrinkage in all cases and symptom improvement in over 90% of the eyes^11^. A quantitative reduction in the cross-sectional conjunctivochalasis area has been demonstrated on anterior segment optical coherence tomography after inferior conjunctival cauterization^28^. Using high-frequency radio-wave electrosurgery to treat conjunctivochalasis, Youm et al reported symptomatic improvement in over 90% of their patients along with a decline in the OSDI scores from 31.7 to 4.2^13^. In our study, mild to moderate conjunctivochalasis was treated with superficial conjunctival cauterization leading to a reduction in the severity of conjunctivochalasis as well as the OSDI scores.

The current study is the first to evaluate the tear film lipid layer thickness before and after surgical treatment of conjunctivochalasis. Previous studies investigated the correlation among lipid layer thickness, dry eye symptoms, and tear film break up time^19^,^20^,^21^,^29^. Severity of dry eye symptoms was negatively correlated with the lipid layer thickness. A thicker lipid layer correlated with a more stable tear film based on tear film break up time^29^. In general, it is believed that a thick lipid layer correlates with a stable tear film^24^. It has been proposed that the lipid layer is repeatedly expanded and compressed between the lid margins during normal blinking^30^. During this phenomenon, meibomian lipid is expressed onto the lipid pool along the lid margin. This lipid is also a reservoir for the lipid layer of the tear film and plays a role in maintaining the stability of aqueous film^31^. The presence of prominent conjunctival folds close to the lid margin and lower tear meniscus in conjunctivochalasis possibly inhibits this phenomenon thereby leading to an inadequate spreading of lipid layer over the ocular surface. This might explain the thinning of the lipid layer in our patients. The lipid layer thickness increased after surgical correction of conjunctivochalasis from 49.6 ± 16.1 nm to 62.6 ± 21.6 nm, which was similar to the ICU of normal subjects (65.0 ± 19.1) measured using LipiView interferometer by Eom *et al.*^20^ Furthermore, the conjunctivochalasis itself could cause ocular irritation. Therefore, reduction of the lax inferior conjunctiva could lead to thickening of the lipid layer as well as an improvement of symptoms and OSDI scores in our patients. The Schirmer’s test results did not change significantly postoperatively indicating that aqueous deficiency was not the major cause of symptoms in our cohort. Likewise, Hara et al reported a significant increase in tear film break up time but no change in Schirmer’s test results after surgery for conjunctivochalasis^27^.

In the current study, there was a significant reduction in corneal thickness and corneal volume after the redundant conjunctiva was excised. Since the precorneal tear film is undetected with Scheimpflug imaging unless fluorescein is added^32^, the change in these parameters may not directly reflect the change in tear film thickness. The difference in corneal thickness and volume before and after the treatment could be related to the structural and functional changes of the corneal epithelium, which is in close proximity to the tear film. Corneal epithelial thickness can be accurately measured using Fourier domain optical coherence tomography^33^. Epithelial thickness was reported to increase in patients with dry eye^34^,^35^. Using the anterior segment optical coherence tomography, central epithelium thickness was reported to be 6.5 μm thicker in dry eye patients compared to the normal subjects^35^. We observed a similar magnitude of variation (5.9 μm) in corneal thickness before and after the surgery. We hypothesized that ocular irritation and dry eye associated with conjunctivochalasis could induce thickening of the corneal epithelium, which reversed after the redundant conjunctiva was excised. It is also known that conjunctivochalasis leads to a delayed tear clearance and accumulation of inflammatory markers in the tear film^36^. Tear matrix metalloproteinase (MMP)-9 levels were elevated in patients with conjunctivochalasis and subsequently decreased after conjunctival resection^5^. MMP-9 has been shown to increase corneal epithelial permeability in animal models of dry eye^37^. This disruption of corneal epithelial barrier could lead to thickening of the epithelium. Normalized tear MMP-9 after treatment of conjunctivochalasis leads to an improvement in epithelial barrier function. Indeed, modification in epithelial thickness has also been suggested as a reactive process in eyes with keratoconus or following corneal refractive surgery^38^,^39^. Unfortunately, we did not measure the corneal epithelial thickness and tear MMP-9 levels in our patients. Nevertheless, the change in corneal thickness was statistically significant before and after the surgery.

Our study highlights the change in tear film and corneal thickness before and after treatment in eyes with conjunctivochalasis. Although the study was limited by a small sample size, our study showed that the tear film lipid layer thickness increased together with an improvement in symptoms after surgical treatment of conjunctivochalasis. The change in corneal thickness and volume observed in our study remains to be elucidated with large sample size and long-term follow-up. It would be prudent to study more patients with different grades of conjunctivochalasis in future studies.

## Methods

This was a prospective interventional study performed at the Hong Kong Eye Hospital between September and November 2014. The Kowloon Central Cluster Research Ethics Committee approved the study protocol. The study adhered to the tenets of the Declaration of Helsinki. All patients gave an informed consent for participation in this study.

Fifteen participants (n = 15 eyes) with no ocular disease except myopia or myopic astigmatism were included as controls in order to assess the repeatability of lipid layer thickness measurements by LipiView interferometer (TearScience Inc., Morrisville, NC, USA). All control subjects were recruited form the Refractive Surgery Clinic of the Chinese university of Hong Kong Eye Clinic. Only one eye of each participant was scanned. Another cohort of 18 consecutive patients (n = 36 eyes) with bilateral symptomatic conjunctivochalasis were enrolled from the outpatient clinics of our hospital from September 2014 to October 2014.

Slit-lamp biomicroscopy followed by lacrimal syringing and probing were performed for all the patients to rule out the presence of lacrimal drainage system obstruction at the time of recruitment. Patients with signs of blepharitis, meibomian gland dysfunction, ocular infection and allergy, nasolacrimal duct obstruction and systemic autoimmune diseases were excluded. Patients using contact lenses or topical corticosteroids or with a history of ocular surgery within the last 6 months were also excluded.

### Investigations

Lipid layer thickness was measured using the LipiView interferometer (TearScience Inc., Morrisville, NC, USA). The unit of measurement is interferometric color units (ICU), which represents the mean interference colors of the tear film. One ICU approximately reflects 1 nm of the lipid layer thickness^21^. For each measurement, the participant was instructed to rest his head on the chin-rest and to blink freely during imaging. The measurement area was digitally set over the cornea, about 1mm above the inferior tear meniscus and manually focused with interface controls. The interferometer was run for its maximum recording duration and the recorded video was automatically analyzed for lipid layer thickness in nanometres (nm) based on recorded interferometric color units.

Ocular Surface Disease Index (OSDI; Allergan Inc., Irvine, CA, USA), a 12-item questionnaire that scores on a scale of 0 to 100, with higher scores representing greater disability, was recorded for all patients. Schirmer’s test, which measures tear production rate, was also performed without any topical anesthesia.

Scheimpflug topography (Pentacam, Oculus Optikgerate GmbH, Wetzlar, Germary) was performed for every patient. Scheimpflug topography captures 100 slit images with a slit depth of 14.0 mm in 2 seconds by rotating along the optical axis from 0 to 360 degrees. It evaluates more than 138,000 true elevation points. The patients were instructed to fixate upon the red central fixation target and keep their eyes wide open just prior to image capture. Once aligned correctly, the digital camera (1.45-megapixel) and slit illumination system (475-nm monochromatic slit of light) automatically rotated around the corneal apex to capture cross-sectional Scheimpflug images of the anterior eye, each separated by 3.6 degrees. Any measurements that were unreliable because of poor alignment, excessive eye movements, or any missing or invalid data were flagged. Scans that were registered as ‘OK’ on the instrument’s Examination Quality Specification were only included for analysis. The same investigator performed all measurements between 10 AM and 4 PM during the course of the study. The maximum keratometry, average keratometry, central corneal thickness, thinnest corneal thickness and corneal volume readings were obtained from a standard printout of the scans.

### Surgical Technique

Bilateral superficial conjunctival cauterization was performed in a single session for every patient. Superficial cauterization was performed under an operating microscope after sterile draping and application of 2% lidocaine to the ocular surface. An assistant held the eye open with a cotton stick applicator at the lower lid while the patient was asked to look into the microscope. The redundant inferior bulbar conjunctiva was lifted up at multiple sites and cauterized with a bipolar cauterizer (Alcon Ltd., Fort Worth, TX, USA) until all the redundant tissue was ablated. Areas close to the limbus were not treated. Postoperatively, topical dexamethasone and chloramphenicol eye drops were used four times daily for one week after the procedure.

All patients were examined 1 month after the surgery. Grading of conjunctivochalasis, OSDI score, Schirmer’s test results and ICU were obtained and compared with the corresponding preoperative values. Topographic parameters including the maximum keratometry value, mean keratometry value, central corneal thickness and corneal volume were also compared before and after the surgery.

### Statistical Analysis

Statistical analysis was performed using Stata Statistical Software version 13.1 (StataCorp LP, College Station, TX, USA) and R version 3.1.0 (R Foundation for Statistical Computing, Vienna, Austria). The measurement repeatability of ICU in normal subjects was assessed through several indicators including, repeatability coefficient (RC), coefficient of variation (CV), and intraclass correlation coefficient (ICC). While RC indicates the mean test-retest measurement differences, CV is the proportion of variation between measurements relative to the mean value of all measurements. ICC is a relative measurement of reliability, in which variation due to measurement error is compared with the variation between subjects. For calculation, RC was defined as 2.77 times the intravisit within-subject standard deviation (Sw). The calculation of Sw was described as the square root of the within-subject mean square of error (the unbiased estimator of the component of variance because of random error) in a 1-way random effects model^40^. The RC, CV (100 × Sw/Overall Mean), and ICC were computed using a customized program developed in R. ICC was interpreted as follows: less than 0.75 represents poor to moderate reliability; 0.75 to 0.90 represents good reliability; greater than 0.90 represents excellent reliability for clinical measures^41^. By setting the 95% confidence interval as 25% on either side of the estimate of Sw, a minimum of 15 subjects (n = 1.96^2^/[2 × 0.25^2^ × (m−1)], where n is the number of subjects and m is the number of observations; m = 3 for this study) would be required in the calculation of test-retest variability. Preoperative and postoperative variables were compared with linear mixed modeling after adjusting for the correlation between fellow eyes. The ICU between eyes of patients and normal subjects were also compared with the same model. The grading of disease severity was compared with Chi-square test. P < 0.05 was considered statistically significant.

## Additional Information

**How to cite this article**: Chan, T. C. Y. *et al.* Change in Tear Film Lipid Layer Thickness, Corneal Thickness, Volume and Topography after Superficial Cauterization for Conjunctivochalasis. *Sci. Rep.* **5**, 12239; doi: 10.1038/srep12239 (2015).

## Figures and Tables

**Table 1:** Interferometric color units and topographic parameters before and after superficial cauterization of conjunctivochalasis.

	**Preoperative**			**Postoperative**			**P**
	**Mean**	**SD**	**Range**	**Mean**	**SD**	**Range**	
**ICU**	49.6	16.1	29–92	62.6	21.6	26–100	<0.001
**Schirmer**	8.1	6.6	1–25	7.4	6.7	0–25	0.399
**OSDI**	31.5	15.0	6.3–68.8	21.5	14.0	4.2–60.4	<0.001
**Kmax**	45.7	2.0	42.0–53.6	45.8	1.9	42.3–51.7	0.568
**Km**	44.0	1.3	41.4–46.7	44.1	1.3	41.5–46.8	0.085
**TCT**	547.9	23.2	489–587	542.2	22.9	481–573	<0.001
**CCT**	553.5	22.8	497–588	547.6	22.6	489–579	<0.001
**CV**	59.7	2.5	53.3–64.8	58.4	2.5	52.0–62.7	<0.001

SD = standard deviation; ICU = interferometric color units; Kmax = maximum keratometry; Km = average keratometry; CCT = central corneal thickness; CV = corneal volume.
